# Conceptualizing the relationship between synthetic cannabinoid use and neuroleptic malignant syndrome

**DOI:** 10.1111/bdi.13503

**Published:** 2024-09-01

**Authors:** Vincent Zhang, Alexis June Wirtz, Anmol Dhingra, Ashar Zahid, Najeeb Hussain

**Affiliations:** ^1^ Department of Psychiatry New Jersey Medical School Newark New Jersey USA

**Key Message**

Synthetic cannabinoids are an increasingly widespread drug of concern. Given their toxicity and newfound ubiquity, psychiatrists need to be able to manage their adverse effects. They have been linked to exacerbation of certain psychiatric conditions, and we highlight a unique case where neuroleptic malignant syndrome was associated with this.

**Learning Points**

1. All patients who require antipsychotics should be screened for concurrent synthetic cannabinoid use, given the propensity of these illicit substances to precipitate muscle injury, kidney damage, and potentially NMS.
2. If both are present, antipsychotics should be discontinued in favor of benzodiazepines and other non anti‐dopaminergic medications.
3. Usage of restraints should also be minimized to reduce further exacerbations of muscle injury.

## 1 CASE PRESENTATION

The patient is a 29‐year‐old female with history of bipolar disorder (multiple past psychiatric admissions) and cannabis/synthetic cannabinoid (SC) use disorder who was recently admitted to an inpatient psychiatric facility for disorganization and agitation. Following this hospitalization, she was discharged on aripiprazole 20 mg daily and lithium 300 mg twice daily. Four days later, she presented to the emergency room following multiple seizure‐like episodes and aggressive behavior. Due to ongoing agitation with violence, patient required multiple doses of droperidol, midazolam, hydroxyzine, and was status post administration of soft restraints. The patient's mother described and recorded three episodes of diffuse jerking that lasted 5 min each, with urinary incontinence and oral frothing. Each episode self‐resolved, and the patient was conversant throughout episodes with no post‐ictal state nor tongue‐biting. Neurology was consulted and concluded that her presentation was not consistent with seizures. The patient subsequently was found to have bradykinesia, rigidity, and a rapid increase in creatine kinase from 310 on arrival to a level of 2428 during evaluation‐ mild neuroleptic malignant syndrome (NMS) was suspected. Her symptoms were thought to be due to use of aripiprazole and droperidol, leading to NMS; however, toxicity from SC's could not be ruled out. Indeed, this was actually suspected, given that the patient had previously been admitted multiple times with similar medication regimens (with higher doses of antipsychotics) without developing NMS. Antipsychotics were discontinued, restraints were avoided as much as possible, and benzodiazepines were instead started for agitation. This resulted in eventual resolution of both her psychiatric and physical symptoms following a short stay in the inpatient unit.

## 2 DISCUSSION

Neuroleptic Malignant Syndrome (NMS) is a well‐known side effect of potentially all antipsychotics, as antagonism of dopamine receptors lead to drops in neurotransmitter activity within dopaminergic pathways.^1^ It has a textbook triad of fever, muscle rigidity, and altered mental status (AMS), but in clinical practice, the majority of cases actually present heterogeneously.^2^ Similarly, synthetic cannabinoids (SC) possess a widely varying drug profile, due to the vast number of derivatives sold. In recent years, SC have become increasingly popular due to their lower cost, easy obtainability and non‐detectability on traditional drug screens.^3^ This is of increasing concern, due to their vastly more unpredictable, severe side‐effects compared to cannabis‐ zero fatalities have been seen with cannabis toxicity, while many have been reported due to SC. Psychiatrically, SC has been show to drastically exacerbate certain conditions including schizophrenia, anxiety, and bipolar disorder. For psychosis in particular, it has been shown that SC use serves as an agonist at cannabinoid type‐1 receptors in the brain, leading to acute increases in dopamine.^4^

NMS and SC use can present very similarly, including increases in CPK, agitation, rigidity, altered mental status, fever etc.^1^, ^4^ For our patient, both likely played a role but which contributed more? It is impossible to tell‐ the former is a clinical diagnosis while SC toxicity cannot be definitively measured.^4^ Regardless, generalizable takeaways can be made. All patients who require antipsychotics (for bipolar disorder, psychosis etc.) should be screened for concurrent SC use. SC use can precipitate muscle injury, leading to elevations in CPK and consequently rhabdomyolysis. We posit this may lead to lowering the threshold for developing NMS following antipsychotic usage, but even if it does not, combining both will almost certainly lead to worse muscle injury.^5^ Instead, benzodiazepines and non‐dopamine acting drugs should be used unless otherwise necessary. Furthermore, for patients who use synthetic cannabinoids, physical restraints should be minimized, as immobilization drastically increases risk of muscle injury. Both of these points are currently not well‐emphasized in the existing literature, but our experience managing patients who present with SC use in an urban, inner‐city hospital points towards this being especially important. Many of our patients who present following SC use have acute psychiatric symptoms (aggression, agitation, altered mental status etc.) that require sedation for both their own safety and that of our staff. Choosing the correct form of management is key to reducing their length of stay and morbidity/mortality. We believe that the increasing ubiquity of synthetic cannabinoids, combined with their potential for adverse reactions with existing medications, highlights the need for further molecular research in this area.

## CONFLICT OF INTEREST STATEMENT

All authors declare that they have no conflicts of interest.

## ETHICS STATEMENT

Informed consent was obtained from the patient, and identifiers were anonymized. Ethics approval from our ethics committee was waived due to the manuscript being a case report.

## Data Availability

The data that support the findings of this study are available on request from the corresponding author. The data are not publicly available due to privacy or ethical restrictions.
